# Genome sequencing and assembly of *Lathyrus sativus* - a nutrient-rich hardy legume crop

**DOI:** 10.1038/s41597-022-01903-4

**Published:** 2023-01-17

**Authors:** Sivasubramanian Rajarammohan, Lovenpreet Kaur, Anjali Verma, Dalwinder Singh, Shrikant Mantri, Joy K Roy, Tilak Raj Sharma, Ashwani Pareek, Pramod Kaitheri Kandoth

**Affiliations:** 1 National Agri-Food Biotechnology Institute, Mohali, 140306 India; 2 Present Address: Division of Crop Sciences, Indian Council of Agricultural Research, Krishi Bhawan, New Delhi, 110001 India; 3 Department of Plant Sciences, Central University of Kerala, Kasaragod, 670320 India

**Keywords:** Plant genetics, Genomics

## Abstract

Grass pea (*Lathyrus sativus*) is a cool-season legume crop tolerant to drought, salinity, waterlogging, insects, and other biotic stresses. Despite these beneficial traits, this crop is not cultivated widely due to the accumulation of a neurotoxin - β-N-oxalyl-L-α, β-diaminopropionic acid (β-ODAP) in the seeds and its association with neurolathyrism. In this study, we sequenced and assembled the genome of *Lathyrus sativus* cultivar Pusa-24, an elite Indian cultivar extensively used in breeding programs. The assembled genome of *Lathyrus* was 3.80 Gb in length, with a scaffold N50 of 421.39 Mb. BUSCO assessment indicated that 98.3% of highly conserved Viridiplantae genes were present in the assembly. A total of 3.17 Gb (83.31%) of repetitive sequences and 50,106 protein-coding genes were identified in the *Lathyrus* assembly. The *Lathyrus* genome assembly reported here thus provides a much-needed and robust foundation for various genetic and genomic studies in this vital legume crop.

## Abstract (summary)

**Table:** 

Measurement(s)	Genome Assembly Sequence
Technology Type(s)	PacBio Sequel System, Illumina sequencing
Factor Type(s)	Lathyrus Pusa-24
Sample Characteristic - Organism	Lathyrus sativus
Sample Characteristic - Environment	leaf
Sample Characteristic - Location	India

## Background & Summary

Grass pea (*Lathyrus sativus*) is a cool-season legume crop cultivated for food mainly in the Indian subcontinent and Ethiopia and as a feed and fodder crop in other parts of the world. *Lathyrus* has various beneficial agronomic traits such as tolerance to drought, salinity, waterlogging, resistance to insects and biotic stresses, and growing well in semiarid and problem soils^1–3^. Furthermore, as a legume crop, it can fix nitrogen. These attributes make it an ideal crop for popularization to sustain agricultural productivity in the changing climatic conditions. Nutritionally, this pulse crop is very rich in proteins, second only to soybean, and provides a balanced amino acid diet in combination with cereals to poor people in countries where it is consumed. It is also a source of L-homoarginine with the potential to increase cardiac health^4^. Moreover, the genus *Lathyrus* belongs to the *Vicieae* tribe, of which important legume crops, *Pisum*, *Lens*, and *Vicia*, are other members. Therefore, research on *Lathyrus* and utilization of its genes underlying valuable agronomic traits like drought resistance, salt resistance, and biotic stress resistance in these closely related genera would be of considerable interest.

Despite many beneficial agronomic and nutritional traits, the major impediment in popularizing this crop is its association with neurolathyrism, characterized by irreversible lower limb paralysis in the affected individuals. Excessive *Lathyrus* consumption for prolonged periods lead to neurolathyrism, which has happened in famine-like situations when the grass pea seeds were consumed as a staple diet. Therefore, one primary research goal in this plant is to understand the mechanisms of neurotoxin β-N-oxalyl-L-α, β-diaminopropionic acid (β-ODAP) accumulation and thereby reduce neurotoxin content in the seeds.

Harnessing the vast diversity of germplasm and the gene pool of *Lathyrus* very much depends on the availability of high-quality genome sequence information. Pusa-24, a popular Indian cultivar, has a β-ODAP content of 0.3–0.6% in the seeds and is the parental plant line used in breeding programs of many low neurotoxin cultivars developed so far^3^. Here we report a high-quality reference assembly of *Lathyrus sativus cv*. Pusa-24.

## Methods

### Genome size estimation

Fresh leaf tissue (~100 mg) from 12–13 days old plants of *Lathyrus*, wheat, and pea (*Pisum sativum*) were taken in a pre-chilled Petri plate kept on ice. Thereafter, 1.5 ml ice-cold Galbraith’s buffer^5^ (45 mM MgCl_2_, 20 mM 3-(N-morpholino) propane sulfonic acid (MOPS), 30 mM sodium citrate, 0.1% (v/v) Triton X-100 and pH 7.0) was added to the plate and chopped the leaves using a new razor blade into very fine slices. Chopping of leaves was performed in four different combinations: *Lathyrus* leaves only, *Lathyrus* + wheat + pea leaves, *Lathyrus* + pea leaves, and *Lathyrus* + wheat leaves. Pea and wheat were used as standard reference samples with known genome sizes. The homogenate was mixed by up and down pipetting without trapping any air bubbles and was filtered through a 40 µm nylon filter. 0.5 ml filtrate was taken into a fresh tube, and 2.5 µl RNase was added and incubated on ice for 15 minutes. To stain the nuclei, propidium iodide (PI) was then added to a final concentration of 50 µg/ml, and samples were kept in the dark for 30 minutes on ice with occasional mixing. Flow cytometry was performed in a BD FACSAria Fusion flow cytometer (BD Biosciences). The genome size of *Lathyrus* was estimated using the known C value parameters of Pea (2 C = 9.09 pg) or wheat (2 C = 34.6 pg) as reference using the formula -

**Sample 2C DNA content** = [(sample G_1_ peak mean)/(Reference G_1_ peak mean)] x Reference 2C DNA content (pg DNA).

### Sample collection, library construction and sequencing

Genomic DNA was extracted from leaves of *L. sativus cv*. Pusa-24 grown at 22 °C, 200 μmol m^−2^ s^−1^ light intensity, 16 /8 hours’ photoperiod and 60% relative humidity using the Qiagen Plant DNA kit as per the manufacturer’s description. The quality and integrity of the extracted DNA were evaluated based on its A260/A280 ratio and its electrophoretic run on an agarose gel. A total of three paired-end (300 bp, 500 bp, and 800 bp insert size), and 3 mate-pair (2–5 Kb, 5–8 Kb, and 8–10 Kb insert size) libraries were generated. The paired-end and mate-pair libraries were generated using the Illumina TruSeq DNA Nano Preparation Kit (Illumina, San Diego, CA, USA), and Nextera Mate Pair Library Preparation Kit (Illumina, San Diego, CA, USA) respectively. All libraries were sequenced on an Illumina HiSeq. 2500 platform following the manufacturer’s instructions. Additionally, for long-read sequencing, libraries were developed using the SMRTbell template preparation kit following the manufacturer’s instructions and sequenced on the PacBio Sequel (I) platform. Finally, ~625 Gb of short-read sequencing raw data and ~85 Gb of long-read sequencing raw data were generated (Tables 1, 2).

**Table 1:** Summary statistics of *Lathyrus* genome raw short-reads.

Sample	Read orientation	Mean read quality (Phred Score)	Number of reads	Number of bases (Mb)	%GC	Mean read length (bp)
Ls_PE_300bp	R1	37.59	932,588,948	139,888.34	38.13	150
	R2	35.85	932,588,948	139,888.34	38.35	150
Ls_PE_500bp	R1	37.04	903,738,101	135,560.72	37.87	150
	R2	33.94	903,738,101	135,560.72	38.41	150
Ls_PE_800bp	R1	37.3	249,534,779	37,430.22	42.49	150
	R2	34.29	249,534,779	37,430.22	42.51	150
Ls_MP_2–5KB	R1	38.35	1,023,903,702	153,585.56	41.38	150
	R2	36.72	1,023,903,702	153,585.56	41.45	150
Ls_MP_5–8KB	R1	38.59	827,875,563	124,181.33	41.02	150
	R2	37.03	827,875,563	124,181.33	41.2	150
Ls_MP_8–10KB	R1	38.12	252,192,293	37,828.84	40.37	150
	R2	36.54	252,192,293	37,828.84	40.64	150

**Table 2:** Summary statistics of *Lathyrus* genome PacBio reads.

	P1 Polymerase Read Bases (Gb)	Polymerase Reads	Polymerase Read Length (mean)	Polymerase Read N50	Insert Length (mean)	Insert N50
1SMRT	9.95	869,031	11,458	20,750	7,971	13,250
2SMRT	3.27	176,904	18,497	28,250	15,442	22,750
3SMRT	4.58	254,727	17,983	27,750	15,097	22,250
4SMRT	4.64	288,146	16,123	25,250	14,083	21,250
5SMRT	7.37	418,468	17,629	28,250	14,632	22,250
6SMRT	9.17	484,211	18,949	29,750	15,036	22,250
7SMRT	12.96	857,699	15,116	26,250	12,303	20,250
8SMRT	11.67	871,091	13,405	23,250	11,338	18,750
9SMRT	10.09	886,870	11,382	19,750	9,999	16,750
10SMRT	11.64	839,611	13,875	24,250	11,554	19,250
Total	85.39	5,946,758	15,441.70	25,350	12,745.50	19,900

### Preprocessing and genome assembly

The raw fastq files were pre-processed before performing assembly. We trimmed the adapters sequences and filtered out reads with an average quality score of less than 30 in any paired-end reads using Trimmomatic v0.36^6^. De novo hybrid assembly was generated using MaSuRCA assembler v4.0.3^7,8^. The cleaned paired-end reads, mate-pair reads, and PacBio long reads were configured as the input data for the hybrid assembly. The assembly was carried out using the default parameters in MaSuRCA. The contig-level assembly covered 3.8 Gb of the genome with a contig N50 value of 78.27 kb (Table 3). Further, the contig-level assembly was scaffolded with *Pisum sativum* as a reference^9^ using the reference-guided scaffolder RaGOO^10^. The scaffolded assembly contained seven chromosome-sized scaffolds and 25404 contigs. The N50 value of the scaffolded assembly was 421.39 Mb (Table 3).

**Table 3:** Summary statistics of the *Lathyrus* genome assembly.

	Contig-level assembly	Scaffolded assembly
**# contigs**	80744	25411
**Total length (Gb)**	3.8	3.805
**GC (%)**	38.32	38.32
**Largest contig (Mb)**	0.504	755.273
**N50 (Mb)**	0.078	421.387
**# N’s per 100 kbp**	0.48	142.94

### Repeat annotation

**Fig. 1:**
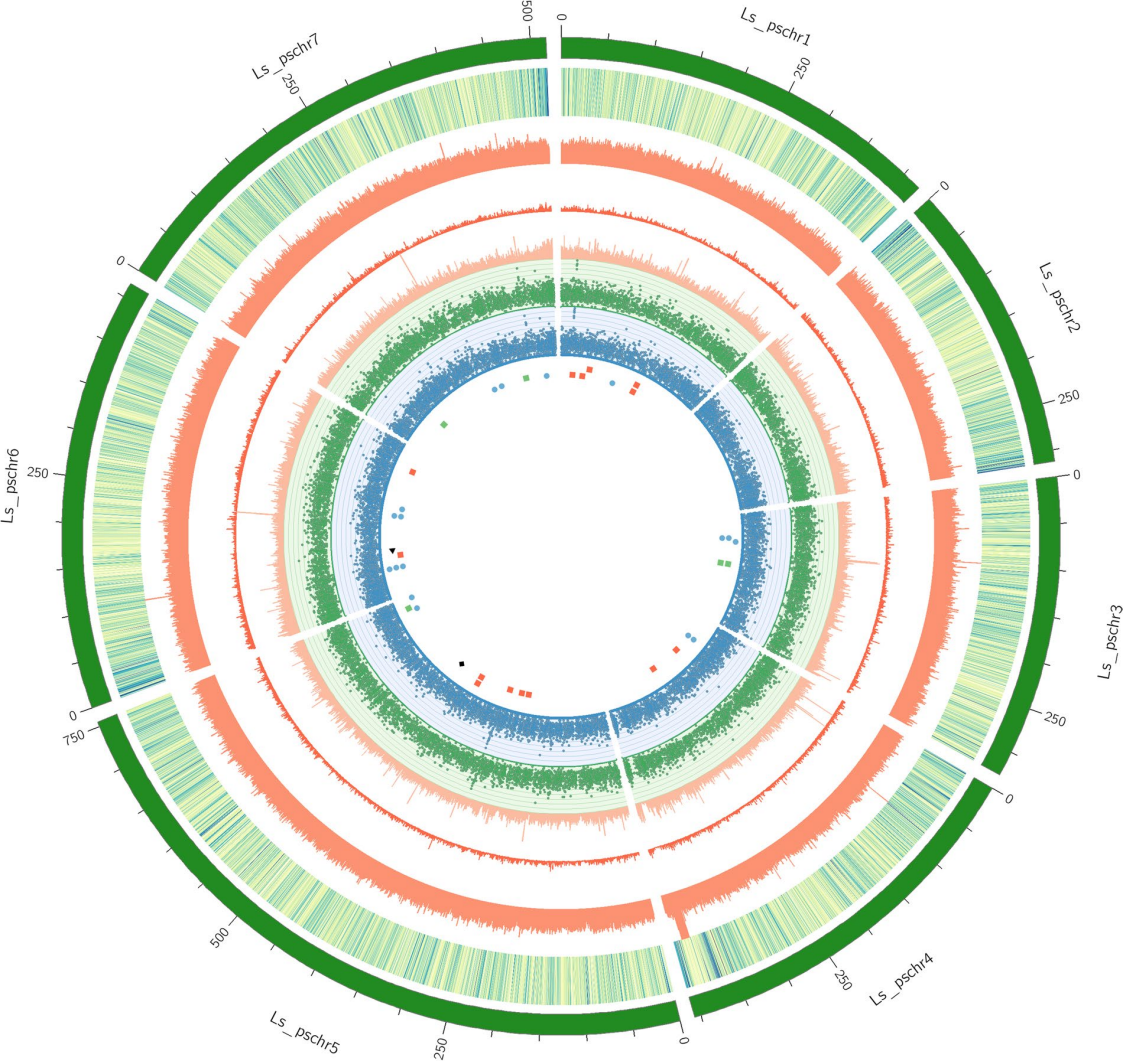
Genome features of *Lathyrus* genome assembly. The circos plot shows, from outside to inside, ideograms of the seven chromosome-sized scaffolds, gene density (blue-green scale), density of DNA transposons, density of LTR retrotransposons, density of simple repeats, Gene expression levels of 4-day and 7-day old seedlings, and position of SSPs on the scaffolds (Red square – Albumins, Blue circle – Legumins, Green square – Lathyrins, Black triangle – Convicillin, and Violet rhombus -Glutelin).

Repetitive regions of the *Lathyrus* genome were identified using RepeatModeler v1.01.11. A de novo repeat library was constructed using RepeatModeler. A combination of the Repbase16^11^ library and the de novo library was then used with RepeatMasker^12^ v4.0.715 to identify repeats in the *Lathyrus* genome. Overall, we identified 3.17 Gb of repetitive sequences, representing 83.31% of the *Lathyrus* genome assembly (Table 4, Fig. 1); of which the long terminal repeat (LTR) elements were the most abundant, accounting for 37.58% of the whole genome.

**Table 4:** Repeat summary statistics of the *Lathyrus* genome assembly.

Description		No. of elements	Occupied length (bp)	% of genome
Retroelements				
	SINEs:	0	0	0
	Penelope:	0	0	0
	LINEs:	27152	16602934	0.44
	CRE/SLACS	1784	647333	0.02
	L2/CR1/Rex	0	0	0
	R1/LOA/Jockey	0	0	0
	R2/R4/NeSL	0	0	0
	RTE/Bov-B	6151	1849178	0.05
	L1/CIN4	19217	14106423	0.37
LTR elements				
	BEL/Pao	0	0	0
	Ty1/Copia	228064	255886719	6.72
	Gypsy/DIRS1	741782	1174154986	30.86
	Retroviral	0	0	0
DNA transposons		73160	56194754	1.48
	hobo-Activator	4678	2841817	0.07
	Tc1-IS630-Pogo	0	0	0
	En-Spm	0	0	0
	MuDR-IS905	0	0	0
	PiggyBac	0	0	0
	Tourist/Harbinger	252	121887	0
Rolling-circles		4045	3135673	0.08
Unclassified		2942024	1556967678	40.92
Total interspersed repeats			3125531853	82.14
Simple Repeats		394692	37580972	0.99
Low complexity repeats		69783	3882912	0.1
			Total Masked %	83.31
			Total genome size	3805271398 bp
			Repeat masked	3170131410 bp

### Gene prediction and annotation

*Ab initio* and homology-based methods along with RNA-seq evidence were combined to predict protein-coding genes using the BRAKER2 v2.1.5^13^ pipeline. For homology-based prediction, protein sequences of seven other legume species (*Cajanus cajan, Cicer arietinum, Glycine max, Medicago sativa, Pisum sativum, Phaseolus vulgaris*, and *Vigna unguiculata*) were downloaded from the Legume federation database (https://www.legumefederation.org/). The RNA-Seq data for *Lathyrus* was derived from a previous study^14^. A total of 50,106 protein-coding genes were predicted, out of which 45,632 were located on the chromosome-sized scaffolds (Fig. 1). The predicted genes were then annotated for their putative biological function by searching against the Uniprot and NCBI nr database. Approximately 96.21% of these genes were functionally annotated by at least one of the databases.

## Data Records

The DNA sequencing data were submitted to the NCBI Sequence Read Archive (SRA) database under the SRA IDs: SRR19732304^15^, SRR18286328^16^, SRR18286326^17^, SRR18286325^18^, SRR18286329^19^, SRR18286327^20^, and SRR18286324^21^, which is associated with the BioProject accession number PRJNA813354. The genome assembly is available at NCBI^22^, and the protein sequences are publicly available at zenodo^23^. Additionally, we have constructed a web portal for the *Lathyrus* genome project (https://lathyrusgenome.nabi.res.in) to benefit the scientific community working on *Lathyrus* and other legume crops. The web portal offers multiple functionalities, including a BLAST search option (against the genome assembly, mRNA, CDS, and protein sequences), an ortholog search-retrieve option, Jbrowse2-based genome visualisation option, and download links to the genome assembly, mRNA, and protein sequences.

## Technical Validation

### Quality assessment of the genome assembly

**Fig. 2:**
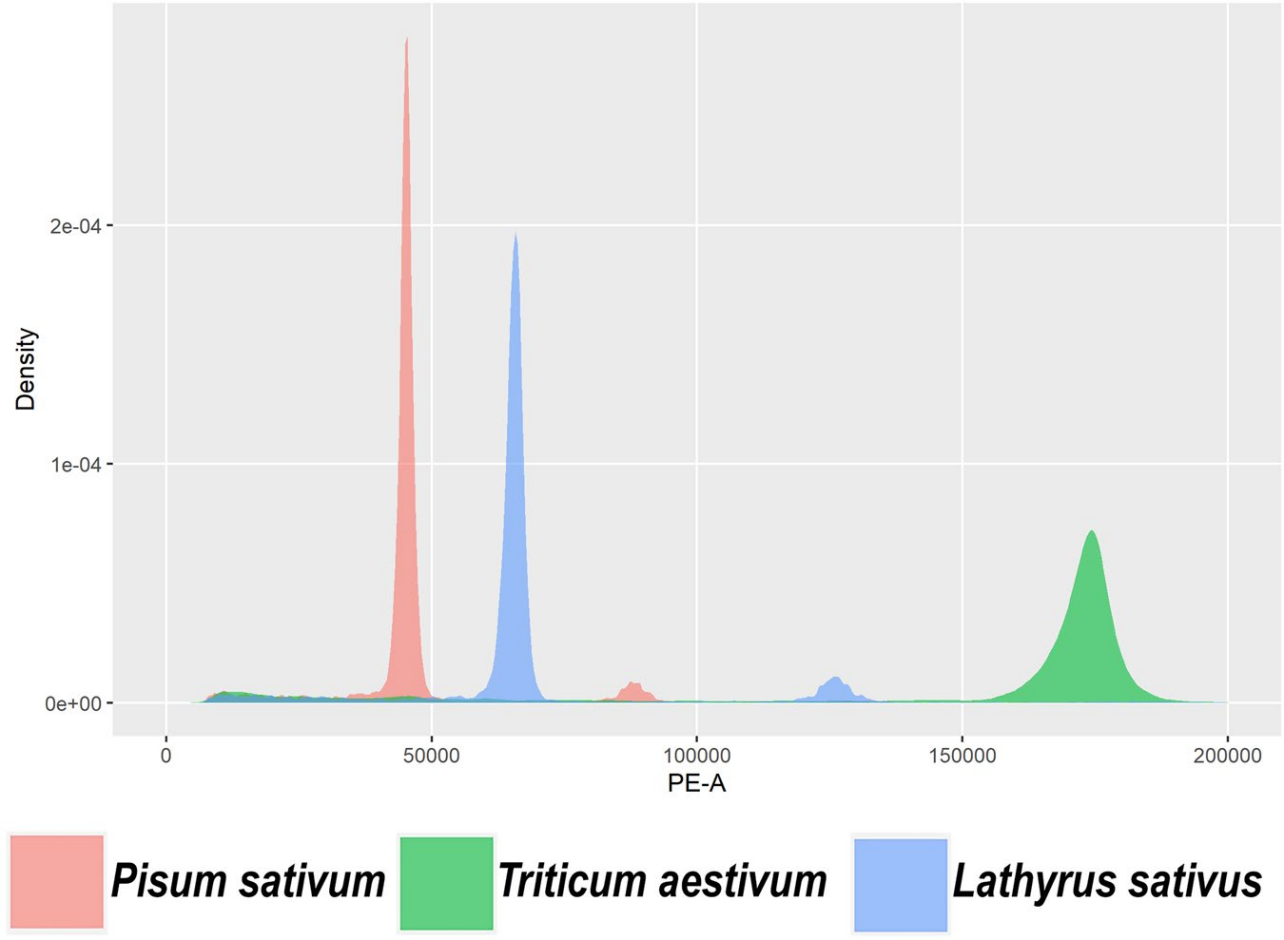
Flow cytometry-based estimation of *Lathyrus* genome size. Propidium iodide fluorescence amplitude (in arbitrary units) is shown against event density (y-axis) which correspond to the G0/G1 DNA for each species. The pea, *Lathyrus*, and wheat nuclei events are denoted by red, blue, and green colors respectively.

The genome size of *Lathyrus* was estimated as 6.62 Gb using Pea (*Pisum sativum*) (4.3 Gb) as reference by flow cytometric analysis (Fig. 2, Table 5). Similar values were obtained when wheat was used as a reference (6.79 Gb, Table 5). The assembly presented here is the first *Lathyrus* genome to be available in the public domain. The contig N50 and scaffold N50 sizes were 78.27 Kb and 421.39 Mb, respectively, with the longest scaffold size 755.27 Mb. A preprint publication describing the draft genome of *Lathyrus* is available; however, the raw and assembled data is not available publicly. We compared the overall assembly statistics of our assembly with that of a draft genome of *Lathyrus* available in the preprint^24^. The draft genome assembly covered 6.2 Gb of the genome; however, it was highly fragmented and had a BUSCO v4^25^ completeness score of 88.4% (Viridiplantae). We carried out BUSCO analysis of both the contig-level assembly and scaffolded assembly to assess the completeness of our assembly and to ascertain if the assembly covered the majority of the gene space. Both the contig-level assembly and scaffolded assembly had a BUSCO completeness score of 98.35% (Viridiplantae) (Table 6), which was higher than the BUSCO scores of the draft genome reported earlier. Additionally, we also subjected both the assemblies to BUSCO analysis with other databases like Eudicots and Fabales, which yielded completeness scores of ~97% and ~96%, respectively (Table 6). Therefore, the gene space coverage in our assembly is adequate and is suitable for various genic analyses involving protein content, β- ODAP metabolism, and drought hardiness.

**Table 5:** Genome size estimation by flow cytometry.

Species	2 C Content	Genome size
*Lathyrus* (Pea as reference)	13.24 ± 0.193	6.62 ± 0.095 Gb
*Lathyrus* (Wheat as reference)	13.58 ± 0.568	6.79 ± 0.284 Gb
Pea (*Pisum sativum*)	9.09	4.54 Gb
Wheat (*Triticum aestivum*)	34.6	17.3 Gb

**Table 6:** Summary of BUSCO analysis of *Lathyrus* genome assembly against Viridiplantae, Eudicots, and Fabales databases.

Viridiplantae				
	Contig-level		Scaffolded	
	Number	%	Number	%
Complete BUSCOs (C)	418	98.35	418	98.35
Complete and single-copy BUSCOs (S)	359	84.47	380	89.41
Complete and duplicated BUSCOs (D)	59	13.88	38	8.94
Fragmented BUSCOs (F)	2	0.47	1	0.24
Missing BUSCOs (M)	5	1.18	6	1.41
Total BUSCO groups searched	425		425	
***Eudicots***				
	**Contig-level**		**Scaffolded**	
	**Number**	**%**	**Number**	**%**
Complete BUSCOs (C)	2256	96.99	2257	97.03
Complete and single-copy BUSCOs (S)	1918	82.46	2009	86.37
Complete and duplicated BUSCOs (D)	338	14.53	248	10.66
Fragmented BUSCOs (F)	19	0.82	19	0.82
Missing BUSCOs (M)	51	2.19	50	2.15
Total BUSCO groups searched	2326		2326	
**Fabales**				
	**Contig-level**		**Scaffolded**	
	**Number**	**%**	**Number**	**%**
Complete BUSCOs (C)	5144	95.86	5149	95.96
Complete and single-copy BUSCOs (S)	4440	82.74	4658	86.81
Complete and duplicated BUSCOs (D)	704	13.12	491	9.15
Fragmented BUSCOs (F)	22	0.41	19	0.35
Missing BUSCOs (M)	200	3.73	198	3.69
Total BUSCO groups searched	5366		5366	

### Gene prediction and annotation validation

**Fig. 3:**
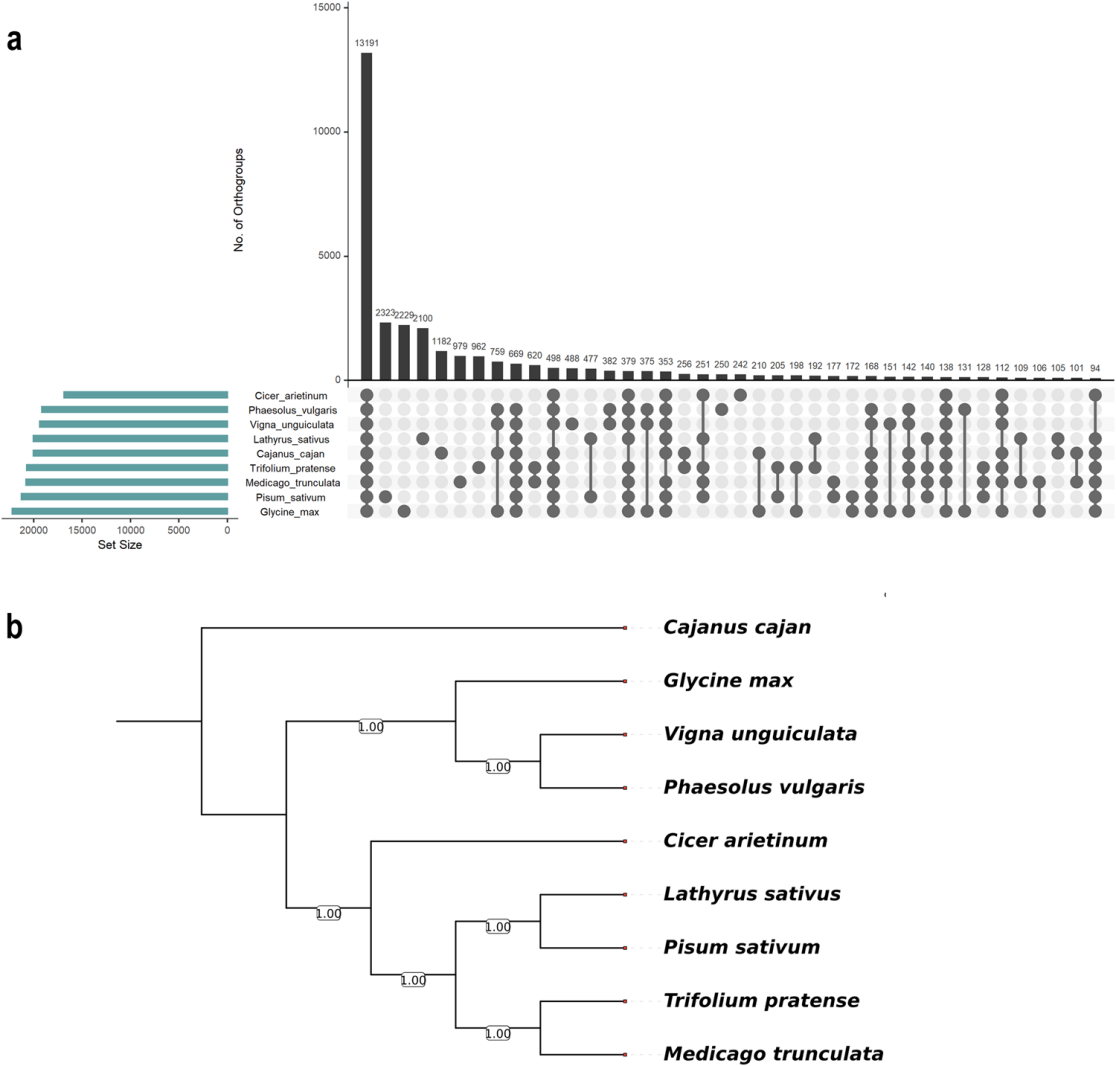
Ortholog analysis of nine legume species including *Lathyrus*. (**a**) A total of 401284 genes from nine species were grouped into 36014 orthogroups. The UpSet plot shows the overlap between orthogroups from each species and the size of overlap as bar charts. (**b**) A maximum likelihood tree representing the phylogenetic relationship between the nine legume species.

Gene models in the *Lathyrus* assembly were predicted using the BRAKER2 pipeline, which used a combination of ab-initio gene prediction, homology-based, and RNASeq evidences. To enhance the quality of the gene prediction, we removed low-quality genes of short length (proteins with fewer than 30 amino acids) and/or exhibiting premature termination. The final gene set consisted of 50,106 genes, which was similar to the other legume species sequenced to date. Also, functional annotation of the predicted gene models indicated that 96.21% of them could be assigned to at least one functional term. Additionally, we also carried out orthology analysis of the *Lathyrus* gene models with the other legume species to validate the predicted genes in the *Lathyrus* assembly. In the orthology analysis, 49,331 genes (94.5%) of *Lathyrus* could be assigned to an orthogroup. A total of 13191 orthogroups contained genes from all the nine legume species, while 2100 orthogroups containing 13840 genes were specific to *Lathyrus* (Fig. 3). Furthermore, 488 single-copy orthogroups identified in the analysis were used to reconstruct a high-confidence phylogenetic tree, which was in concordance with previous studies that determined the phylogenetic relationship among the legumes (Fig. 3).

**Fig. 4:**
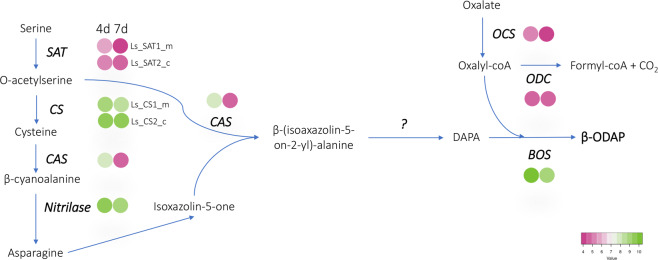
Biosynthesis of β-ODAP in *Lathyrus sativus*. The biosynthetic pathway of β-ODAP along with the genes/enzymes catalysing each step in the reaction is shown. The colored circles denote the expression levels of the corresponding gene in 4-day and 7-day old *Lathyrus* seedlings.

Further, to confirm the validity and quality of gene prediction of the *Lathyrus* assembly, we searched for genes that contribute to β-ODAP biosynthesis in the *Lathyrus* genome. Since β-ODAP, an endogenous non-protein amino acid, is present exclusively in *Lathyrus* and not in the other legume species, identification of the biosynthetic genes of this non-protein amino acid in the current genome assembly will further affirm the quality of the gene prediction. β-ODAP biosynthesis is believed to occur in the mitochondria and chloroplasts and originate from precursors - asparagine and serine^26^. We identified most of the known genes associated with this pathway, viz. Serine O-acetyltransferase (SAT), Cysteine synthase (CS), cyanoalanine synthase (CAS), nitrilase, β-ODAP synthetase (BOS), oxalyl-CoA synthetase (OCS), and oxalate decarboxylase (ODC). The *Lathyrus* genome has five copies of the Serine O-acetyltransferase (SAT) gene; however, only two are expressed during the 4- and 7-day old seedlings (Fig. 4). Cysteine synthase (CS) is encoded by a multigene family in plants that includes cyanoalanine synthase (CAS) and other related enzymes. The *Lathyrus* genome encodes eight CS genes, out of which one may be a CAS. Previous studies reported only five CS isoforms, including a CAS^26^. Therefore, these results indicate that the predicted gene set of the *Lathyrus* genome is complete and of high quality and can be used for various gene discovery studies.

## Data Availability

All software used in this work are in the public domain, with parameters described in the Methods section. If no parameters were mentioned for a software tool, default parameters were used as suggested by the developer.
